# The Effects of Carbon Content on the Anisotropic Deformation Mechanism of Boron Carbide

**DOI:** 10.3390/ma11101861

**Published:** 2018-09-29

**Authors:** Jun Li, Lisheng Liu, Shuang Xu, Jinyong Zhang, Yuanli Wu

**Affiliations:** 1 Hubei Key Laboratory of Theory and Application of Advanced Materials Mechanics, School of Science, Wuhan University of Technology, Wuhan 430070, China; whut_lijun@126.com (J.L.); m15871438618@163.com (Y.W.); 2 State Key Laboratory of Advanced Technology for Materials Synthesis and Processing, Wuhan University of Technology, Wuhan 430070, China; liulish@whut.edu.cn (L.L.); jyzhang@whut.edu.cn (J.Z.); 3 Institute of Advanced Material Manufacturing Equipment and Technology, Wuhan University of Technology, Wuhan 430070, China

**Keywords:** boron carbide, first-principles method, carbon content, anisotropic deformation mechanism

## Abstract

The effects of carbon content on the mechanical properties and deformation mechanisms of boron carbides were investigated by first-principles calculations, based on the density functional theory. The B_12_–CBC (13.33 at % C) and B_10_$C_{2}^{P}$–CC (28.75 at % C) were studied and then compared with the deformation of regular B_11_C^P^–CBC (20.0 at % C). The results show the B_10_$C_{2}^{P}$–CC, which has the lowest carbon content, has the highest strength and hardness as well as the lowest toughness. With the increase of carbon content, the rhombohedral symmetry will be broken and the three-atoms chains will be replaced by diatomic carbon chains. These changes may have an influence on their anisotropic deformation mechanisms. For the B_12_–CBC, the destruction of icosahedra without bending three-atom chains causes structural failure for compression along the *c* axis; while for compression along the *a* axis, new B–B bonds are formed, causing an unrecoverable deformation; then it is gradually destroyed until full destruction. For the B_10_$C_{2}^{P}$–CC, the anisotropic deformation mechanism is not obvious. For both loading directions, the breakage of B–C^P^ bonds causes the stress to drop, suggesting that the structure is beginning to be destroyed. Finally, the icosahedra are fully destroyed, resulting in structural failure.

## 1. Introduction

Boron carbide is characterized by many outstanding properties, such as thermal stability, extreme abrasion resistance, high hardness, and low density, which make it a promising material for a wide range of engineering applications, such as in semiconductors, refractory processes, abrasive power, and body armors [1,2,3,4,5,6]. The crystal structure of boron carbide is very complicated [7,8,9], it consists of 12-atom icosahedra located at vertices of a rhombohedral unit cell and the 3-atom chains lying along the main diagonal axis. This structure of boron carbide can also be described in terms of a hexagonal lattice, in which case the [0001] axis of the hexagonal lattice is related to the body diagonal of the primitive rhombohedral unit cell. There are two types of crystallographic sites, “polar” and “equatorial”, within an icosahedron. Atoms at polar sites link the icosahedra together, while atoms at equatorial sites connect to chains. In addition, boron carbide is generally regarded to have R$\bar{3}$m symmetry. However, this can be only true for a subset of the available atomic arrangements, since the substitution of carbon atoms into icosahedra may cause a distortion of the rhombohedral lattice, thereby reducing the crystalline symmetry [8].

Similar to other ceramic materials, the mechanical properties and deformation mechanisms of boron carbide strongly depend on its chemical compositions, microstructure, and fabrication processes [1,2,10,11,12,13,14,15]. In particular, depending on the synthesis conditions, boron carbide has a relatively broad composition range, from 8 to 20 at % C, with varying distributions of carbon (C) and boron (B) atoms into icosahedra and chains to form thermodynamically stable solid solutions, resulting in a complex phase diagram [2,16]. However, due to the complex structure of boron carbide, as well as the similarities of atomic form factors for X-ray diffraction [17] and nuclear scattering cross-sections (^11^B and ^12^C) for neutron diffraction [9] between C and B atoms, it is a challenge to distinguish C from B atoms and identify their exact atomic positions at any specific carbon content experimentally. Several first-principle calculations have predicted two stable forms at 13.33 and 20 at % C, corresponding to B_13_C_2_ and B_12_C_3_ stoichiometries, respectively, represented by B_12_–CBC and B_11_C^P^–CBC, where superscript p represents the polar site [8,14,18]. Since the maximum at % C of boron carbide is still an unsolved question and debatable among the research [1,2,12,19], Ektarawong et al. [11] investigated the thermodynamic stability of carbon-rich boron carbide at different compositions, ranging from 20 to 33.33 at % C, using first-principles calculations. They concluded that apart from B_4_C, the only carbon-rich boron carbide with 28.75 at % C, denoted by B_2.5_C, is thermodynamically stable under high pressures with respect to B_4_C as well as pure B and C phases. The atomic configuration of the B_2.5_C is represented by B_10_$C_{2}^{P}$–CC, where icosahedra C^P^ atoms occupying the polar sites of the icosahedra without forming C^P^–C^P^ bonds and C–C denotes a diatomic carbon chain. They also suggested a possible route for experimental synthesis of B_2.5_C as well as a fingerprint for its characterization from the simulations of x-ray powder diffraction.

The lattice constants, atomic bonding, mechanical properties, and deformation behaviors of boron carbide have been suggested to change with the carbon content [1,2,15,20,21]. Aselage et al. [20] determined the lattice constants of boron carbide by power x-ray diffraction for samples with compositions between ~7.7 and 20.5 at % C, and established the relationship between lattice constants and carbon contents. They concluded that the *a* parameter increases almost linearly with the decrease of carbon content. The expended lattice constants maybe result from the difference in atom radii between C and B atoms. Further, neutron powder diffraction data [9] have shown that the chain bond length of boron carbide at about 13 at % C is reduced 2–3% compared to that in boron- and carbon-rich materials. In addition, mechanical properties of boron carbide have been shown to change with the carbon content [1,2,15,21]. Nanoindentation measurements [21] have demonstrated that the hardness and elastic modulus of boron-rich boron carbides decrease with the increase of boron content, except for B_10.2_C. Domnich et al. [2] and Taylor et al. [15] compared the elastic properties of boron carbide with different carbon contents. They concluded that the toughness of boron carbide decreases as the increase of boron content. In addition, the deformation mechanisms of boron carbide with different carbon contents is not the same [16,17,18,19]. An et al. [22,23,24] studied the deformation mechanism of boron carbides with different carbon contents under shear deformation, corresponding to B_12_C_3_ (B_4_C) and B_13_C_2_ and B_14_C stoichiometries. The deformation mechanism of B_4_C involves two steps. They found a unique ‘plastic’ deformation before failure, in which the B–C bond between neighboring icosahedra breaks to form a reactive carbene, and the 3-atom chains have bent sufficiently for the carbene to form new bonds with B atoms in the chain center. Then, the icosahedra begins to be destroyed, and finally structural failure occurs [22]. For B_13_C, the structure deforms continuously without bending the 3-atom chain and then the structure fails suddenly [23]. For B_14_C (B_12_–CBB), which is a boron-very-rich boron carbide, the brittle failure arises from the interaction between B_12_ icosahedra and the bent C–B–B chains [24]. Taylor et al. [15] also studied the effect of stoichiometry on the deformation behaviors of boron carbide under loading. They suggested that within all structures, the structural failure of boron carbides results from a sudden bending of the 3-atom chains under *c* axis (the direction of 3-atom chains) compression. However, in our previous studies [25], we found that under *c* axis compression, the deformation mechanism of B_4_C is the formation of new B-B bonds between icosahedra and chains and not the chain bending. Thus, the deformation mechanism of boron carbide with different carbon contents proposed by Taylor et al. [15] may not be reasonable. Furthermore, since boron carbide has strong anisotropic elasticity, interatomic bonding, and deformation behaviors [25,26,27], it is necessary to investigate the anisotropic deformation mechanism for boron carbide and then further investigate the effects of carbon content on the deformation behaviors of boron carbides.

Although there are extensive experimental and theoretical studies about boron carbides, the anisotropic deformation mechanisms for boron carbide with different carbon contents, particularly those under uniaxial compressions, remain a mystery. Furthermore, since boron carbide powder are most likely a mixture of boron carbide configurations, to fully explain the failure process of boron carbide materials, it is necessary to understand the anisotropic deformation mechanism of boron carbide with different carbon contents. However, this has not been well understood. Understanding the anisotropic deformation mechanism for boron carbide with different carbon contents can also help us to identify ‘soft’ configurations that may initiate failure in boron carbide structures under impact loading.

In this article, two stable forms with 13.33 and 28.75 at % C, corresponding to B_6.5_C and B_2.5_C stoichiometries, respectively, by B_12_–CBC and B_10_$C_{2}^{P}$–CC were taken as examples for boron-rich boron carbide and carbon-rich boron carbide, respectively. Then the two stable configurations were used to investigate the mechanical behaviors and deformation processes of boron carbide by first-principles methods, based on density functional theory. The deformation mechanisms were compared with that of regular stable boron carbide B_4_C (B_11_C^P^–CBC) to understand the influence of carbon content. For each stable configuration, elastic properties as well as stress-strain responses under hydrostatic and uniaxial compressions were studied. To examine the anisotropic deformation mechanism, it is essential to investigate the deformation behaviors of boron carbide with different carbon contents under *c* axis and *a* axis compressions. The rest of this paper is organized in four sections. In Section 2, details about the computational methods are given. In Section 3 and Section 4, we present the results and discussion about the mechanical properties and anisotropic deformation mechanisms of boron carbides with different carbon content. Some main conclusions are suggested in Section 5. 

## 2. Methods 

The density functional theory (DFT) has been considered to be one of the most accurate methods for the computation of physical properties and deformation mechanisms of solids [28,29,30]. The DFT and the plane-wave projector augmented wave (PAW) method with the local density approximation (LDA) functional, as implemented in the Vienna ab initio Simulation Package (VASP) periodic code [31,32,33], were used for all periodic calculations. To ensure accuracy and efficiency, tests were made before calculations to determine the number of k-points and the cutoff energy required. According to the test results, the plane-wave energy cutoff and the Monkhorst-Pack k-point mesh was set to be 800 eV and 5 × 5 × 5, respectively. The convergence criteria were set to be 1 × 10^−6^ eV energy difference for solving the electronic wave function and 1 × 10^−3^ eV/Å force for geometry optimization. And the symmetry of the crystal structure was maintained during the calculations. These parameters could provide excellent convergence on the energy, force, stress, and structural parameters of boron carbides.

During the geometry optimization, all internal atomic coordinates, volume, and cell shape were fully relaxed and then the optimized models were obtained. To evaluate the mechanical properties of the two configurations, elastic constants (Cij), bulk modulus (B), shear modulus (G), Young’s modulus (E), passion ratio (v), Pugh’s ductility index (B/G), Vickers hardness (H_V_), and anisotropy indexes (A^U^) were examined. The elastic constants Cij are related to the second derivative of the total energy with respect to strain, and they were derived from the stress-strain response as a function of various cell distortions from equilibrium configuration. The elastic stability of boron carbide was examined using Born stability criteria [11,15], as shown in Equation (1). Then, the other mechanical properties could be evaluated [34,35,36].

C11 − |C12| > 0,
(C11 + C12) × C33 − 2 × (C13) × 2 > 0,
(C11 − C12) × C44 − 2 × (C14) × 2 > 0,
C44 > 0 (1)

To investigate the anisotropic deformation mechanism for boron carbide, we applied the uniaxial compressive strains along the *a* axis and *c* axis, while allowing full structure relaxation of the other five strain components. Then, the residual stresses after relaxation were less than 0.1 GPa. Thus, under the uniaxial compression, the other two axes were expanded during the relaxation. At each deformation step, a small increment (a 1% compressive strain) was applied sequentially to the structure relaxed in the previous step. To perform the hydrostatic compression, hydrostatic pressure was also imposed on these models with a 20 GPa increment, and then the total energy and volume strain were evaluated. Since the compressive strain was constrained in the deformation, the stress of the system could become negative after the structure changes or fails.

## 3. Results

### 3.1. Structural and Elastic Properties

**Figure 1:**
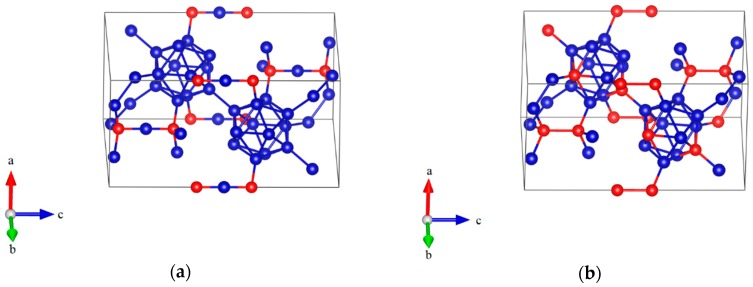
The atomic structures of boron- and carbon-rich boron carbide with hexagonal lattice: (**a**) B_12_–CBC configuration; (**b**) B_10_$C_{2}^{P}$–CC configuration (Blue = B atoms; red = C atoms).

In the present work, two optimized models with hexagonal lattice for B_6.5_C and B_2.5_C were constructed, as shown in Figure 1a,b, respectively. In Figure 1a, the B_6.5_C structure, corresponding to B_12_–CBC, with 13.33 at % C is indicted as the most plausible structure for boron-rich boron carbide, in which B_12_ denotes the icosahedron and the 3-atom linear chain is C–B–C [14]. The B_12_–CBC structure has the R$\bar{3}$m space group. As shown in Figure 1b, the B_2.5_C structure, represented by B_10_$C_{2}^{P}$–CC, at 28.57 at % C is predicted to be the most stable configuration for carbon-rich boron carbide, where the icosahedral C^P^ atoms residing in the polar sites of the icosahedra without C^P^–C^P^ bonds and C–C represent a diatomic carbon chain [11]. As mentioned in the introduction, the symmetry of B_10_$C_{2}^{P}$–CC structure is not R$\bar{3}$m because the C atoms substitute into B_12_ icosahedra, breaking its rhombohedral symmetry.

The optimized equilibrium lattice parameters of B_12_–CBC and B_10_$C_{2}^{P}$–CC structures are listed in Table 1 and then compared with those of the B_11_C^P^–CBC structure. Since a B atom has a slightly larger atomic radius than a C atom, the lattice parameters of boron carbide slightly expanded with the increase of boron content. It is consistent with experimental observation [2,21]. To confirm the thermodynamic stability of boron carbides, we computed the formation energy with respect to their constituent elements, given by α-boron and graphite. The computed formation energy for B_12_–CBC, B_11_C^P^–CBC and B_10_$C_{2}^{P}$–CC is −73.67, −116.56 and −72.64 meV/atom, respectively. It means that these structures are thermodynamically stable crystal structures and can be synthesized by conventional methods. The value of formation energy can also represent the stability of the compounds. Thus, B_11_C^P^–CBC presumably lies on the convex hull of the B–C system because of its smallest formation energy.

Then, elastic properties were calculated for each structure. The calculated elastic constants of boron carbides are shown in Table 2. The calculated elastic constants of these structures satisfied the Born stability criteria listed in Equation (1), suggesting that they are mechanically stable. In addition, the elastic properties of boron carbide have been shown to change with the carbon content. The isotropic polycrystalline elastic modulus, including bulk modulus (B), shear modulus (G), and Young’s modulus (E), were estimated using the Voigt–Reuss–Hill (VRH) method [36], as shown in Table 3. The mechanical moduli are increased by increasing the carbon content in the structure. Thus, the general trend is that the strength of boron carbide decreases with lower carbon concentrations. Then, according to the relation between Vickers hardness (H_V_) and shear modulus proposed by Chen et al. [35], i.e., H_V_ = 0.151 G, which was shown to hold for many materials, the hardness of boron carbides can be estimated. As shown in Table 3, the hardness of B_10_$C_{2}^{P}$–CC is the highest among these structures because of its high shear modulus, indicating that the hardness of boron carbide increases with the carbon content.

Generally, the Pugh’s ductility indexes (B/G) is frequently used to indicate the ductility of compounds [24,37]. It is supposed that for brittle compounds, B/G is smaller than 1.75 (for diamond B/G = 0.8) and for metallic compounds, B/G is greater than 1.75 (for Al B/G = 2.74) [37]. As shown in Table 3, the Pugh’s ductility index for B_12_–CBC, B_11_C^P^–CBC and B_10_$C_{2}^{P}$–CC are all smaller than 1.75, suggesting that these structures are brittle materials. Among these structures, the Pugh’s ductility indexes for B_10_$C_{2}^{P}$–CC is the smallest, indicating that the toughness of B_10_$C_{2}^{P}$–CC is the lowest among these boron carbides. Moreover, anisotropy index A^U^, which is a new universal anisotropy indexes given by Ranganathan et al. [34], can be calculated by A^U^ = 5G_V_/G_R_ + B_V_/B_R_ − 6, and could be used to characterize the anisotropy in elasticity of boron carbides. A value of unity means that the crystal exhibits isotropic properties, while values otherwise represent varying degrees of anisotropy. As shown in Table 3, although B_12_–CBC has the highest symmetry among these structures, it also suffers from the strongest anisotropy. This character can also be found in titanium borides compounds [37].

### 3.2. Stress-Strain Relationship

#### 3.2.1. Hydrostatic Compression

**Figure 2:**
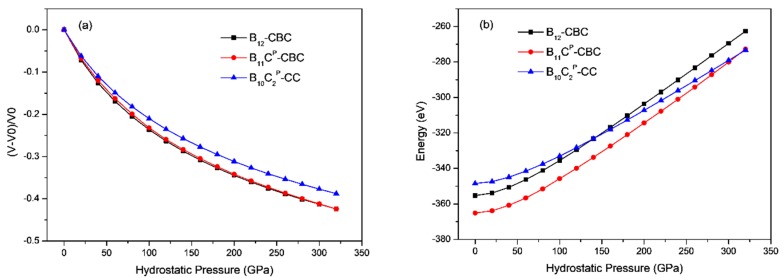
(**a**) The volume strain vs. hydrostatic pressure (**b**) the total energy vs. hydrostatic pressure in B_12_–CBC, B_11_C^P^–CBC and B_10_$C_{2}^{P}$–CC configurations. The volume strain and total energy varies continuously.

The results of hydrostatic compression on B_12_–CBC, B_11_C^P^–CBC and B_10_$C_{2}^{P}$–CC are shown in Figure 2, where the volume strain and total energy are plotted with the hydrostatic pressure. As displayed in Figure 2a, under the same hydrostatic pressure, the volume strain for B_10_$C_{2}^{P}$–CC is the smallest among these structures due to its highest bulk modulus. In Figure 2a,b, the volume strain and total energy vary continuously and smoothly in all models even when the hydrostatic pressure increases to 320 GPa.This agrees with previous theoretical calculations [27,38].

#### 3.2.2. Uniaxial Compressions

**Figure 3:**
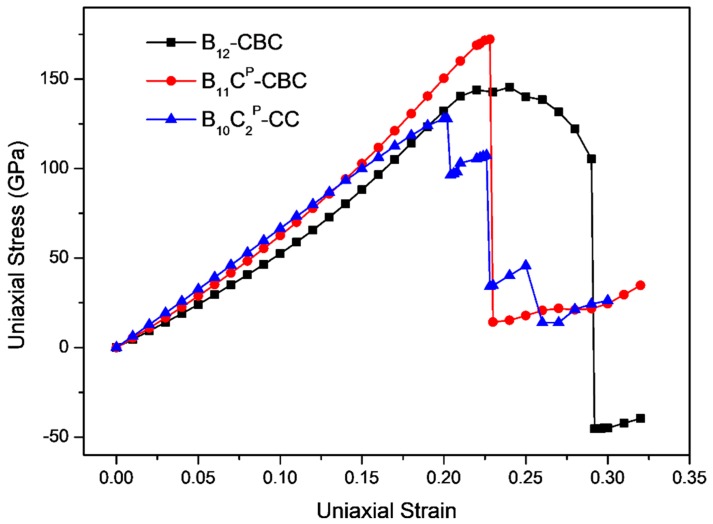
The stress-strain curves for on B_12_–CBC, B_11_C^P^–CBC and B_10_$C_{2}^{P}$–CC configurations under *c* axis uniaxial compression.

**Figure 4:**
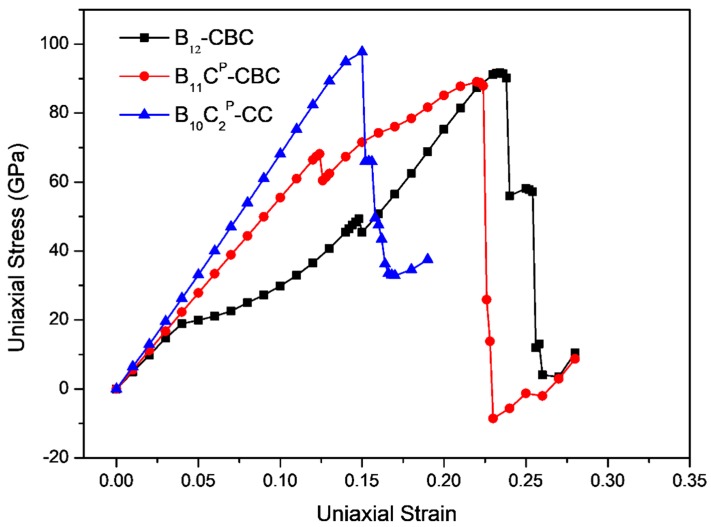
The stress-strain relationships for on B_12_–CBC, B_11_C^P^–CBC and B_10_$C_{2}^{P}$–CC configurations under *a* axis uniaxial compression.

The stress-strain curves for uniaxial compression along the *c* axis and the *a* axis of B_12_–CBC and B_10_$C_{2}^{P}$–CC structures are displayed in Figure 3 and Figure 4, respectively, and compared with the results of the regular B_11_C^P^–CBC crystal [25]. In Figure 3 and Figure 4, there are several abrupt points in the stress-strain curves for these structures. To further investigate the deformation behaviors related to these abrupt points, the strain increment was changed from 1% to 0.2% in these regions. As shown in Figure 3, the maximum stress for on B_12_–CBC and B_10_$C_{2}^{P}$–CC are 145.46 GPa and 127.87 GPa, respectively, which is lower than the strength limit of B_11_C^P^–CBC (172.18 GPa).

In Figure 3, the stresses initially increase almost linearly up to the maximum stress, indicating that these structures are uniformly resistant to the deformation. Then, for the B_12_–CBC structure, the stress varies continuously from its maximum stress (145.46 GPa at *ε* = 0.24) to 105.38 GPa with *ε* = 0.29 without structural changes. Finally, at *ε* = 0.292, an abrupt drop in stress to −45.37 GPa occurs, suggesting that the B_12_–CBC structure is destroyed. However, for the B_10_$C_{2}^{P}$–CC structure, beyond the maximum stress, there is a first sudden stress drop to 96.46 GPa with *ε* = 0.204 then the stress increases continuously. As the strain increases, the stress ultimately drops to 34.21 GPa at *ε* = 0.228, and then appears to fluctuate, indicating that the B_10_$C_{2}^{P}$–CC structure is under destruction. Under *c* axis compression, the critical failure strain for B_12_–CBC, B_11_C^P^–CBC and B_10_$C_{2}^{P}$–CC is 0.292, 0.23 and 0.228, respectively, suggesting that the B_10_$C_{2}^{P}$–CC is the most brittle structure among these configurations.

For uniaxial compression along *a* axis, as displayed in Figure 4, the maximum stress for B_10_$C_{2}^{P}$–CC is 97.79 GPa, which is higher than that of B_12_–CBC (91.67 GPa) and B_11_C^P^–CBC (89.04 GPa). For the B_12_–CBC structure, as the strain increases to 0.148, the stress increases continuously, indicating that the structure deforms elastically. At *ε* = 0.15, similarly to B_11_C^P^–CBC structure, there is a small stress fluctuation before reaching the maximum stress. Then the stress monotonically increases until it reaches the maximum stress of 91.67 GPa at *ε* = 0.234. After that, the stress decreases slightly from 91.67 GPa to 90.20 GPa at *ε* = 0.238. At *ε* = 0.24, the stress drops suddenly to 55.98 GPa. Finally, the stress drops again to 11.89 GPa at *ε* = 0.256. While for B_10_$C_{2}^{P}$–CC structure, unlike the B_12_–CBC and B_11_C^P^–CBC structures, the stress increases almost linearly and monotonically until reaching the maximum stress of 97.79 GPa at *ε* = 0.15. Beyond the point of maximum stress, the stress decreases generally. At compressive strain *ε* = 0.152, there is a sudden drop in stress to 65.97 GPa. Then, the stress drops again to 49.65 GPa at *ε* = 0.158. Finally, the stress ultimately drops to a minimum value 32.88 GPa with *ε* = 0.17. Under *a* axis compression, the critical failure strain for B_12_–CBC, B_11_C^P^–CBC and B_10_$C_{2}^{P}$–CC is 0.256, 0.23 and 0.17, respectively, indicating that the toughness of the B_10_$C_{2}^{P}$–CC structure is the lowest among these structures under *a* axis compression.

The above results indicate that the mechanical behaviors and deformation processes of B_12_–CBC and B_10_$C_{2}^{P}$–CC structures for uniaxial compression along *c* axis and *a* axis are very different, mainly because of the anisotropy of the B_12_–CBC and B_10_$C_{2}^{P}$–CC structures. To explain the underlying anisotropic deformation mechanism, structural changes and the isosurface of the electron localization function (ELF) in B_12_–CBC and B_10_$C_{2}^{P}$–CC are further studied. It is a reliable way to analyze the lone pair formation and covalent bonding [39,40,41].

### 3.3. Anisotropic Deformation Mechanism

#### 3.3.1. *c* Axis Compression

**Figure 5:**
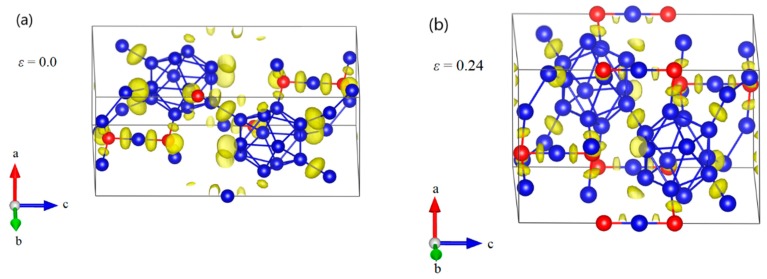

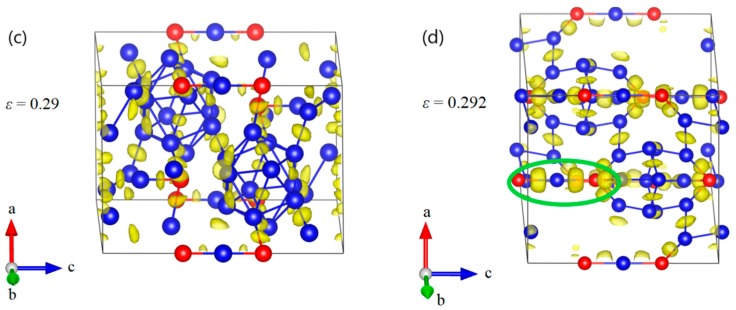
The structural changes and electron localization function (ELF) at various critical strains for B_12_–CBC structure under *c* axis compression: (**a**) the undeformed structure; (**b**) the structure at *ε* = 0.24 relating to the maximum stress; (**c**) the structure at *ε* = 0.29 before structural failure, where the chains are still straight and the icosahedra are intact; (**d**) the structure at *ε* = 0.292 where the structure fails because the icosahedra are fully destroyed without bending the C–B–C chains (Blue = B atoms; red = C atoms).

To understand the deformation behaviors of B_12_–CBC under c axis compression, structural changes in the B_12_–CBC structure were examined, as displayed in Figure 5. Figure 5a shows the undeformed structure of B_12_–CBC. As the compressive strain increases to 0.29, the structure deforms elastically. The C–B–C chains are still straight and the icosahedra are intact, but deformed slightly, as shown in Figure 5b,c. At compressive strain *ε* = 0.292 (Figure 5d), the B_12_ icosahedra are fully destroyed without bending the C–B–C chains, leading to a sudden drop in stress to a negative value (Figure 3). Thus, the main reason for the structural failure of B_12_–CBC structure is the destruction of icosahedra.

**Figure 6:**
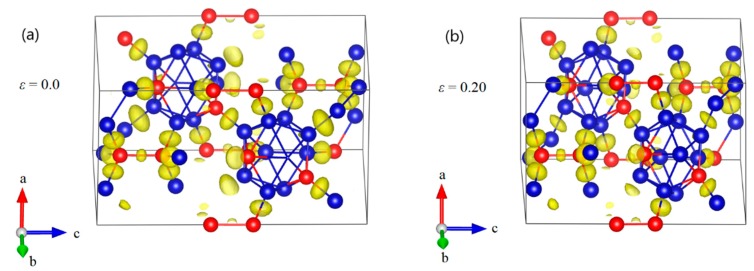

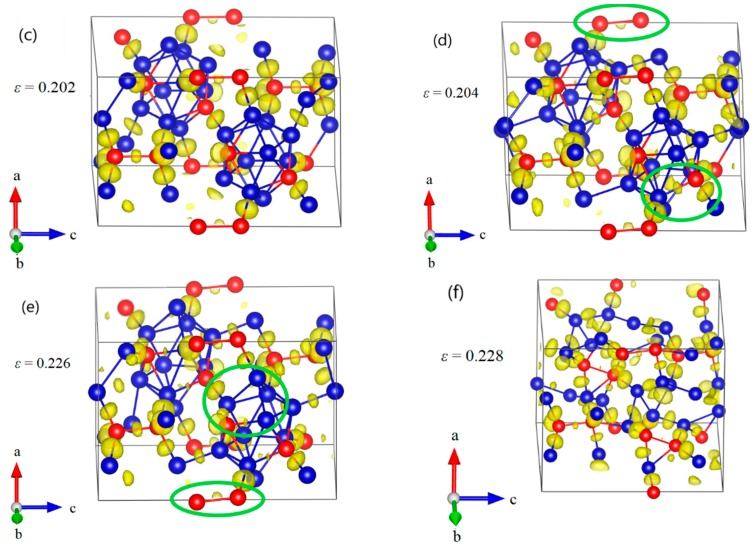
The structural changes and ELF at various critical strains for B_10_$C_{2}^{P}$–CC structure under *c* axis uniaxial compression: (**a**) the initial structure; (**b**) the structure at *ε* = 0.20 relating to the maximum stress; (**c**) the structure at *ε* = 0.202 before the first stress drop, where the icosahedra are intact without breaking any bonds; (**d**) the structure at *ε* = 0.204 after the first stress drop, where the B–C^P^ bonds within icosahedra are broken; (**e**) the structure at *ε* = 0.226 before structural failure; (**f**) the structure at *ε* = 0.228 where the structure fails because of the fully destruction of icosahedra (Blue = B atoms; red = C atoms).

However, the deformation behaviors for the B_10_$C_{2}^{P}$–CC structure are more complicated than that of B_12_–CBC, as shown in Figure 6. Figure 6a displays the undeformed structure of B_10_$C_{2}^{P}$–CC. As the compressive strain increases to 0.202, the structure uniformly resists the elastic deformation and the icosahedra are intact without breaking any bonds (Figure 6b,c). At compressive strain *ε* = 0.204 (Figure 6d), one of the B–C^P^ bonds within the icosahedron stretches from the original 1.666 Å to 2.302 Å, indicating that the B_10_$C_{2}^{P}$–CC icosahedra begin to be destroyed. At the same time, the diatomic carbon chains are no longer parallel to the c axis but there is no new bond formed. Thus, the first sudden stress drops in the B_10_$C_{2}^{P}$–CC structure mainly results from the breakage of B–C^P^ bonds within icosahedra. Then, the stress monotonically increases from 96.46 GPa to 107.45 GPa at *ε* = 0.228 without totally destroying the B_10_$C_{2}^{P}$–CC icosahedra. The icosahedra are highly disordered but still identifiable (Figure 6e). As displayed in Figure 6f, the B_10_$C_{2}^{P}$–CC icosahedra are fully destroyedabove compressive strain of 0.26, causing the stress to fluctuate up and down without any patterns. The B_10_$C_{2}^{P}$–CC structure is fully destroyed.

#### 3.3.2. *a* Axis Compression

**Figure 7:**
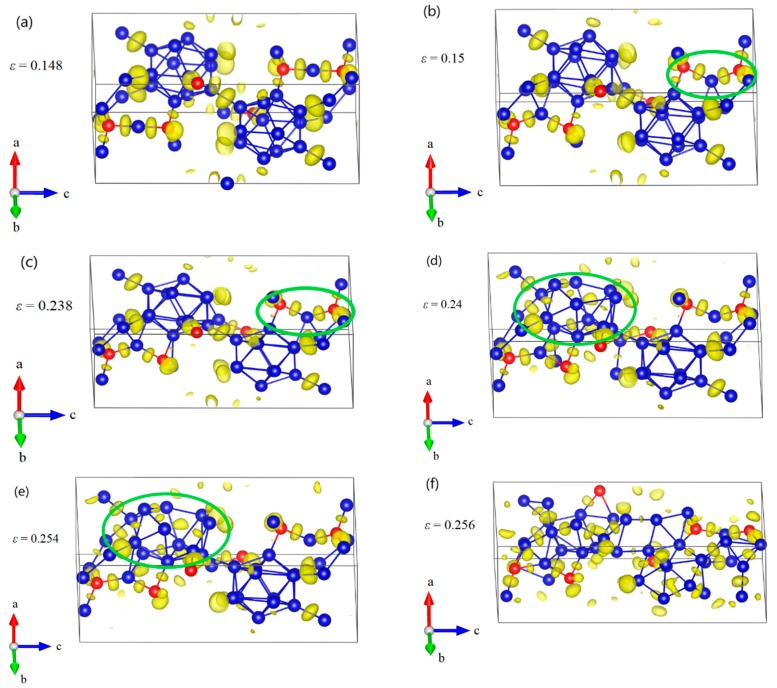
The structural changes and ELF at various critical strains for B_12_–CBC structure under *a* axis uniaxial compression: (**a**) the structure at *ε* = 0.148 where it uniformly resists the elastic deformation and the C–B–C chains are almost straight; (**b**) the structure at *ε* = 0.15 where some new B–B bonds are formed, leading to a small stress fluctuation; (**c**) the structure at *ε* = 0.238 before the first significant stress drop where the icosahedra are identifiable and intact; (**d**) the structure at *ε* = 0.24 after the first stress drop, where one of the icosahedra are broken. (**e**) the structure at *ε* = 0.254 before structural failure; (**f**) the structure at ε = 0.256 where the icosahedra and the structure are fully destroyed (Blue = B atoms; red = C atoms).

**Figure 8:**
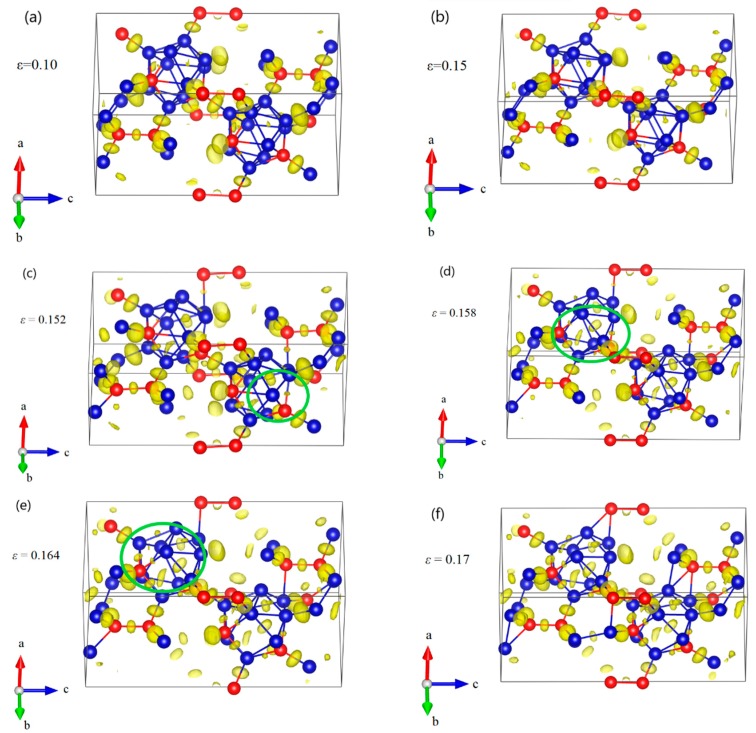
The structural changes and ELF at various critical strains for B_10_$C_{2}^{P}$–CC structure under *a* axis uniaxial compression: (**a**) the structure at *ε* = 0.10 in elastic deformation stage; (**b**) the structure at *ε* = 0.15 relating to the maximum stress, where the icosahedra are intact; (**c**) the structure at *ε* = 0.152 after the first stress drop, where one of the B–C^P^ bonds within the icosahedron is broken; (**d**) the structure at *ε* = 0.158 after the second stress drop, where all B–C^P^ bonds within icosahedra are broken; (**e**) the structure at *ε* = 0.164, where the icosahedra are gradually damaged; (**f**) the structure at *ε* = 0.17 where the structure fails due to the fully destruction of icosahedra (Blue = B atoms; red = C atoms).

**Figure 9:**
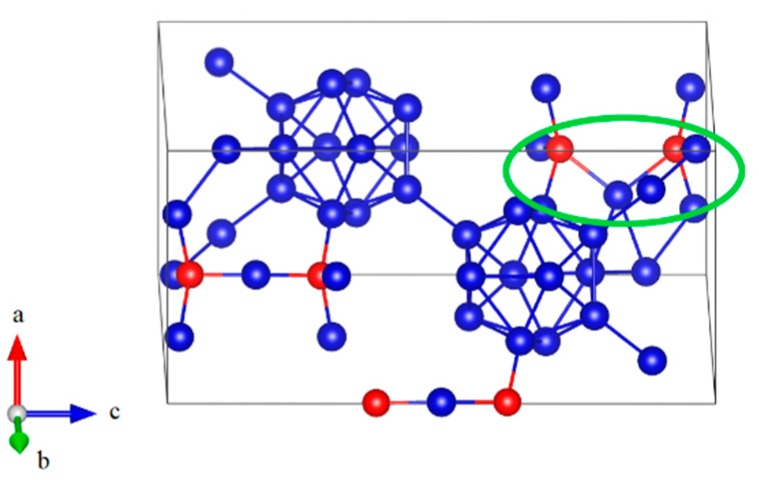
The structure after unloading from *ε* = 0.15 for B_12_–CBC structure under *a* axis uniaxial compression, where the 3-atom chain remain bending, implying an unrecoverable deformation occurs (Blue = B atoms; red = C atoms).

Since B_11_C^P^–CBC has strong anisotropic elasticity, interatomic bonding, and deformation behaviors [25,26,27], it is also necessary to understand the compression deformation mechanisms for B_12_–CBC and B_10_$C_{2}^{P}$–CC under different loading directions. The structural changes of B_12_–CBC and B_10_$C_{2}^{P}$–CC under *a* axis uniaxial compression were examined, as displayed in Figure 7 and Figure 8. As shown in Figure 7a, the B_12_–CBC structure uniformly resists the elastic deformation until the compressive strain reaches 0.148. The C–B–C chains are almost straight and the icosahedra are intact. Then at *ε* = 0.15, the B atoms in the chain center form new B–B bonds with the B atoms in neighboring icosahedra, causing a small stress fluctuation, as displayed in Figure 7b. In this process, the angle of chains decreases suddenly from 179.9° to 129.2°, while the icosahedra remain intact and deform slightly. To further explain the deformation behavior of the B_12_–CBC structure at *ε* = 0.15, unloading calculations were performed. As illustrated in Figure 9, the 3-atom chains remain bending after unloading from *ε* = 0.15, implying an unrecoverable deformation occurs on B_12_–CBC structure at this strain level. After that, the stress monotonically increases to the maximum value of 91.67 GPa at *ε* = 0.234. Then, at compressive strain *ε* = 0.238 (before the first stress drop), there is a slight decrease in stress from 91.67 GPa to 90.2 GPa. However, the icosahedra are still identifiable and intact, as displayed in Figure 7c. In Figure 7d, the B_12_–CBC structure is partly broken, so that only one icosahedron is in destruction, causing a first sudden drop in stress to 55.98 GPa at *ε* = 0.24. Then, as the compressive strain further increases to 0.254 (before the second stress drop), the stress varies continually to 57.18 GPa, without totally destroying the structure (Figure 7e). Finally, as shown in Figure 7f, the icosahedra and the structure are fully destroyed at compressive strain *ε* = 0.256, causing the stress to fluctuate up and down without any patterns.

The structural changes of B_10_$C_{2}^{P}$–CC structure under *a* axis compression are displayed in Figure 8. As the compressive strain increases to 0.15, the icosahedra are intact but slightly deformed (Figure 8a,b). Then one of the B–C^P^ bonds within the icosahedron increases from the original 1.792 Å to 2.639 Å at *ε* = 0.152, causing a sudden drop in stress to 65.97 GPa (Figure 8c). The B_10_$C_{2}^{P}$–CC icosahedra begin to be destroyed. At *ε* = 0.158 (Figure 8d), all B–C^P^ bonds within icosahedra are broken, leading to the second sudden stress drop to 49.65 GPa. In this process, the icosahedra are not intact, but still identifiable. After that, in Figure 8e, the icosahedra are gradually destroyed, resulting in the monotonic decrease in stress (Figure 4). Finally, the structure fails, due to the fully destruction of icosahedra, as shown in Figure 8f.

## 4. Discussion

In our work, three stable configurations of boron carbide, represented by B_12_–CBC (13.33 at % C), B_11_C^P^–CBC (20.0 at % C) and B_10_$C_{2}^{P}$–CC (28.75 at % C), were used to investigate the effects of carbon content on the mechanical properties and anisotropic deformation mechanisms of boron carbide. The results have shown that atomic structure and carbon content have an obvious effect on the structural properties and mechanical properties of boron carbides. As displayed in Table 1, the lattice parameters of boron carbides slightly expanded with the increases of boron content, due to the different atomic radius between B and C atoms. Since the substitution of C atoms in the icosahedra induces a small distortion, only the B_12_–CBC structure has the R$\bar{3}$m space group. In addition, the elastic properties of boron carbide have been found to change with the carbon content. As shown in Table 3, the strength and the hardness of boron carbide increase with the increasing of carbon content. The Pugh’s ductility index implies that all structures exhibit brittle behaviors, and the toughness of B_10_$C_{2}^{P}$–CC is lowest among these structures. Moreover, B_10_$C_{2}^{P}$–CC also suffers from the weakest anisotropy, because of its lowest anisotropy indexes. These results agree well with previous experimental and theoretical studies [1,2,15,21].

For all structures under hydrostatic compression, continuous changes in volume strain and total energy were observed up to a very high hydrostatic pressure of 320 eV. However, when compressed along *c* axis or *a* axis direction, the structures underwent massive structural changes and final failure. Under uniaxial compressions, the strength is significantly overestimated in comparison with other experiments [5,42]. One possible reason is that the perfect crystal structure of boron carbides was considered in our work. Thus, the role of defects such as point defects, crystal impurity and microcracks on the deformation mechanism of boron carbide was omitted. But it is almost impossible to obtain such perfect crystal structures in experiments. Another reason is the use of ionic relaxation at zero temperature in the first-principles calculations. Computational results have also suggested that the maximum stress along the *c* axis is much larger than that of the *a* axis, indicating that the strength of the boron carbide crystal is higher along the [0001] direction. However, it should be noted that C_11_ is higher than C_33_ for the B_12_–CBC, B_11_C^P^–CBC and B_10_$C_{2}^{P}$–CC structural configurations. This apparent contradiction may be in terms of internal relaxation of the boron carbide lattice under external stress [43], and the instability of atom chains under perpendicular loading. Although the B_10_$C_{2}^{P}$–CC structure has the largest elastic moduli, the breakage of B–C^P^ bonds within icosahedra at 0.204 strain releases its stress under *c* axis compression, decreasing its maximum strength below that of the B_11_C^P^–CBC and B_12_–CBC structures. And the results also imply that the toughness of B_10_$C_{2}^{P}$–CC which has the smallest Pugh’s ductility index, is the lowest among these configurations, because of its lowest failure strain.

In addition, the effects of carbon content on the anisotropic deformation mechanism for boron carbide were further examined. For the B_12_–CBC structure with 13.33 at % C, under *c* axis compression, the destruction of icosahedra without bending C–B–C chains is the main mechanism for structural failure; while under *a* axis compression, the new B-B bonds between chains and icosahedra are formed, resulting in a small stress fluctuation before reaching the maximum stress. In the process, an unrecoverable deformation occurs. Then, the structure is gradually destroyed, causing the two sudden drops in stress. Finally, the icosahedra and the structure are fully destroyed. For the B_11_C^P^–CBC structure with 20.0 at % C [25], the deformation mechanism is related to the formation of new B-B bonds between chains and icosahedra for *c* axis compression and the destruction of icosahedra for *a* axis compression. We should note that for the B_11_C^P^–CBC structure, there is also a small stress fluctuation in the elastic deformation stage results from abrupt bending of C–B–C chains without forming new bonds. The different deformation mechanisms between B_12_–CBC and B_11_C^P^–CBC structures maybe results from the C atom in the icosahedra, which breaks the rhombohedral symmetry (R$\bar{3}$m) of B_11_C^P^–CBC structure.

However, for B_10_$C_{2}^{P}$–CC structure with 28.75 at % C, whose anisotropy indexes A^U^ is the lowest, the deformation mechanism for compression along the *a* axis and *c* axis directions is slightly different. Under *c* axis compression, the breakage of one of the B–C^P^ bonds with the icosahedron results in the first stress drop in the B_10_$C_{2}^{P}$–CC structure. And then the structure can sustain the further compressive deformations without totally destroying the B_10_$C_{2}^{P}$–CC icosahedra. Finally, the full destruction of B_10_$C_{2}^{P}$–CC icosahedra leads to structural failure. While under *a* axis compression, the breakage of one of the B–C^P^ bonds within the icosahedron causes the first stress drop. Then all B–C^P^ bonds within icosahedra are broken, leading to the second sudden stress drop. After that, the icosahedra are gradually destroyed until full destruction, leading to structural failure. The above results imply that the B–C^P^ bond within icosahedra is the weakest bond of the B_10_$C_{2}^{P}$–CC structure. Since diatomic carbon chains are more stable than three-atom chains, the B–C^P^ bond within icosahedra of B_10_$C_{2}^{P}$–CC structure is easier to be broken than that of B_11_C^P^–CBC structure. Maybe this is the reason for its lowest strength along *c* axis compression and its lowest toughness.

## 5. Conclusions

In this work, first-principles methods were used to examine the effects of carbon content on the mechanical properties and anisotropic deformation mechanism of boron carbides. In our work, the B_12_–CBC (13.33 at % C) and B_10_$C_{2}^{P}$–CC (28.75 at % C) structures were studied and then compared to the deformation processes of the B_11_C^P^–CBC structure (20 at % C). The elastic constants, bulk modulus, shear modulus, Young’s modulus, passion ratio, Pugh’s ductility index, Vickers hardness, and anisotropy indexes were examined. The results show that the mechanical properties of boron carbide change with carbon content. The general trend is that the strength and hardness of boron carbides increases with higher carbon concentrations. But with the increase of carbon content, the toughness of boron carbides decreases. The anisotropy of B_10_$C_{2}^{P}$–CC is the lowest among these structures.

For all structures, continuous changes in volume strain and total energy were observed under hydrostatic compression. However, for uniaxial compression, the structures underwent massive structural changes and final failure. The effects of carbon content on the anisotropic deformation mechanism for boron carbides were further examined by comparing their deformation behaviors under *a* axis and *c* axis uniaxial compressions. For the B_12_–CBC structure with 13.33 at % C, under *c* axis compression, the structural failure is caused by the destruction of icosahedra without bending of chains; while under *a* axis compression, the formation of new B–B bonds between chains and icosahedra results in a small stress fluctuation before reaching the maximum stress, causing an unrecoverable deformation. After that, the icosahedra in B_12_–CBC are destroyed in turn until the structure is fully destroyed. The anisotropic deformation mechanism is different from that of B_11_C^P^–CBC structure (20.0 at % C) because it has R$\bar{3}$m symmetry without C atoms in the icosahedra. For the B_10_$C_{2}^{P}$–CC structure with 28.75 at % C, the anisotropic deformation mechanism is not obvious. The breakage of B–C^P^ bonds is the main reason for the stress drops in B_10_$C_{2}^{P}$–CC, suggesting that the B_10_$C_{2}^{P}$–CC structure began to be destroyed. Finally, the full destruction of B_10_$C_{2}^{P}$ icosahedra leads to structural failure. With the increase of carbon content, the three-atom chains were replaced by diatomic carbon chains. Since diatomic carbon chains are more stable than three-atom chains, the breakage of the weakest B–C^P^ bond within icosahedra causes the B_10_$C_{2}^{P}$–CC structure to begin to be destroyed. Maybe this is the reason for its lowest strength along *c* axis compression and lowest toughness.

Real boron carbide powder is likely a mixture of different structural configurations. The deformation mechanism will be far more complex. The present simulations explain the effects of carbon content on the anisotropic deformation mechanism for boron carbide clearly. In-depth understanding in the deformation behaviors is the basis for increasing deformation resistance in boron carbides and for designing and synthetic boron carbide composites with high-strength and high-toughness.

## Figures and Tables

**Table 1:** Lattice parameters of boron carbides.

Formula	Configuration	% C	a (Å)	b (Å)	c (Å)	α (°)	β (°)	γ (°)
B_6.5_C	B_12_–CBC	13.33	5.60	5.60	11.96	90.00	90.00	120.00
B_4_C ^1^	B_11_C^P^–CBC	20.00	5.53	5.53	11.91	92.03	87.97	119.89
B_2.5_C	B_10_$C_{2}^{P}$–CC	28.57	5.42	5.40	11.01	90.00	88.88	119.83

^1^ Ref. [25].

**Table 2:** The calculated elastic constants Cij (GPa) of boron carbides using the stress-strain method.

Formula	Configuration	% C	C_11_	C_12_	C_13_	C_14_	C_33_	C_44_
B_5.6_C ^1^	Experiment	15.2	542.8	130.6	63.5	/	534.5	164.8
B_6.5_C	B_12_–CBC	13.33	526.7	142.5	83.1	10.5	465.3	99.2
B_4_C	B_11_C^P^–CBC	20.00	580.4	135.1	73.2	15.8	547.6	170.5
B_2.5_C	B_10_$C_{2}^{P}$–CC	28.57	658.1.	99.6	94.2	−20.1	642.9	301.1
B_2.5_C ^2^	B_10_$C_{2}^{P}$–CC	28.57	620	90	75	−23	605	290

^1^ Ref. [26], ^2^ Ref. [11].

**Table 3:** The bulk modulus B (GPa), shear modulus G (GPa), Young’s modulus E (GPa), Poisson ratio (v), Pugh’s ductility index (B/G), Vickers hardness (H_V_) and anisotropy indexes (A^U^) of boron carbides calculated using the elastic constants, with the Voigt–Reuss–Hill (VRH) approximations applied for the evaluation of mechanical moduli.

Formula	Configuration	% C	B			G			E	v	B_H_/G_H_	H_V_	A^U^
			B_V_	B_R_	B_H_	G_V_	G_R_	G_H_					
B_5.6_C ^1^	Experiment	15.2	/	/	237	/	/	195	460	0.18	1.22	29.4	/
B_6.5_C	B_12_–CBC	13.33	237.3	238.1	237.7	158.8	141.2	150.0	371.6	0.24	1.58	22.7	0.62
B_4_C	B_11_C^P^–CBC	20.00	255.0	252.9	253.9	207.7	198.2	202.9	480.7	0.18	1.25	30.6	0.25
B_2.5_C	B_10_$C_{2}^{P}$–CC	28.57	279.1	277.8	278.4	285.9	285.7	285.8	638.8	0.12	0.97	43.2	0.01

^1^ Ref. [26].
